# Supplementary material for: The intelligent evaluation model of the English humanistic landscape in agricultural industrial parks by the SPEAKING model: From the perspective of fish-vegetable symbiosis in new agriculture
Source: PLoS One. 2025 Jul 16;20(7):e0325332. doi: 10.1371/journal.pone.0325332 (PMC12266420; doi:10.1371/journal.pone.0325332)
Supplement: S1 File — (ZIP) [file pone.0325332.s001.zip › Code Description.docx]

**Import Necessary Libraries**

- At the beginning of the code, the relevant libraries are imported, mainly including:
  - torch and torch.nn: Used to build deep learning models, define neural network structures, and perform tensor operations.
  - transformers: Provides pre-trained BERT models and tokenizers.
  - torch.utils.data: Supports the construction and loading of datasets.
  - numpy: Used for numerical computations.
  - sklearn.metrics: Used for accuracy calculation in model evaluation.

Define the Dataset Class: TranslationDataset

- **Function:** Converts input text and labels into a format that can be accepted by the model.
- **Main Methods:**
  - __init__: Initializes the dataset, including input text, labels, tokenizer, and maximum length.
  - __len__: Returns the size of the dataset.
  - __getitem__: Returns a single data entry based on index, including:
    - input_ids (the tokenized ID sequence of the input text).
    - attention_mask (indicating the position of valid tokens).
    - label (the corresponding label, numericalized).
- **Key Points:**
  - The tokenizer.encode_plus method is used for tokenizing the text, supporting maximum length, padding, and truncation to ensure uniform input format.

Define the Model Class: TranslationEvaluationModel

- **Function:** Uses a pre-trained BERT model for translation evaluation.
- **Main Components:**
  - BertModel: A pre-trained BERT model loaded from transformers, responsible for generating text features.
  - classifier: A linear layer that maps the BERT output features to the label space (such as an 8-class classification task).
- **Forward Method:**
  - Takes input_ids and attention_mask as input.
  - Uses BERT to generate contextual features (pooler_output).
  - Passes the features through the classifier to generate predicted logits.

Training Function: train_model

- **Function:** Implements a single round of training for the model.
- **Inputs:**
  - model: The model to be trained.
  - dataloader: Data loader providing training data.
  - optimizer: The optimizer that adjusts model parameters.
  - criterion: The loss function used to calculate model error.
  - device: Specifies the device (CPU/GPU).
- **Main Logic:**
  - Sets the model to training mode.
  - Iterates over each batch of data:
    - Resets the optimizer gradients.
    - Performs forward propagation to compute predictions.
    - Uses the cross-entropy loss function to calculate loss.
    - Performs backward propagation to update model parameters.
  - Returns the average training loss.

Evaluation Function: evaluate_model

- **Function: Evaluates the model’s performance on the validation or test set.**
- **Inputs:**
  - model: The model to be evaluated.
  - dataloader: Data loader providing validation or test data.
  - device: Specifies the device for evaluation.
- **Main Logic:**
  - Sets the model to evaluation mode (no gradient computation).
  - Iterates over each batch of data:
    - Performs forward propagation to compute predictions.
    - Uses torch.argmax to get predicted class labels.
    - Collects all predictions and true labels.
  - Uses accuracy_score to calculate accuracy.

Main Program Section

- **Parameter Initialization:**
  - PRETRAINED_MODEL_NAME: Selects the pre-trained BERT model (bert-base-uncased).
  - MAX_LENGTH: Sets the maximum length of input sequences (128).
  - BATCH_SIZE: The batch size for each training iteration (16).
  - NUM_EPOCHS: The number of training epochs (3).
  - NUM_LABELS: The number of categories in the classification task (8 classes).
  - LEARNING_RATE: Learning rate (2e-5).
- **Data Preparation:**
  - Defines example input texts and corresponding labels.
  - Uses the TranslationDataset class to generate dataset objects.
  - Creates data loaders (DataLoader) to dynamically load and shuffle batch data.
- **Model Construction:**
  - Initializes TranslationEvaluationModel.
  - Specifies the device as GPU or CPU.
- **Optimizer and Loss Function:**
  - Uses the AdamW optimizer, suitable for large-scale language models.
  - Uses the cross-entropy loss function (nn.CrossEntropyLoss).
- **Training Loop:**
  - Iterates over the number of epochs, calling train_model.
  - Outputs the average loss for each epoch.
- **Model Evaluation:**
  - Calls evaluate_model to compute and output accuracy.

Code Features

- **Modular Design:** The code divides functionality such as data processing, model definition, training, and evaluation into independent modules for easier maintenance and expansion.
- **Scalability:** Supports various translation evaluation tasks (e.g., text, image, or speech inputs).
- **High Availability:** Compatible with both GPU and CPU, making it suitable for large-scale data training.
