# Supplementary material for: The intelligent evaluation model of the English humanistic landscape in agricultural industrial parks by the SPEAKING model: From the perspective of fish-vegetable symbiosis in new agriculture
Source: PLoS One. 2025 Jul 16;20(7):e0325332. doi: 10.1371/journal.pone.0325332 (PMC12266420; doi:10.1371/journal.pone.0325332)
Supplement: S1 File — (ZIP) [file pone.0325332.s001.zip › Dataset Description.docx]

The WMT Chinese-English Machine Translation Training Corpus is a large-scale and high-quality parallel corpus for Chinese-English translation, widely used for training and evaluating machine translation models, particularly for bidirectional Chinese-English translation tasks. This dataset integrates parallel sentence pairs from multiple authoritative sources, covering various language domains and scenarios, and is suitable for a wide range of research needs in deep learning models. It is one of the most important resources in the field of machine translation.

**1. Data Sources**

The corpus comes from diverse and authoritative sources, including the following main datasets:

- **ParaCrawl**
  Automatically crawled Chinese-English web documents, covering multiple domains. The data is aligned using parallel corpus techniques. Although the dataset is large, it contains noise.
- **News-Commentary**
  A Chinese-English parallel corpus mainly based on news and commentary texts. The structure is clear, and it is focused on the news domain, making it suitable for news translation tasks.
- **Wiki-Titles**
  Title data from Wikipedia in multiple languages. It has high alignment quality and is suitable for short-text translation tasks.
- **UN Parallel Corpus**
  Official document translations from the United Nations, providing high-quality aligned Chinese-English texts for formal scenarios, with strong professionalism.
- **WikiMatrix**
  A corpus aligned using language models from multilingual Wikipedia documents, suitable for cross-lingual text comparison tasks.
- **China Conference on Machine Translation (CCMT)**
  A Chinese-English translation benchmark dataset provided by the China Conference on Machine Translation, with strict data selection, making it an important benchmark for machine translation research.

**2. Data Scale**

The WMT Chinese-English Machine Translation Training Corpus contains around 25 million pairs of Chinese-English parallel sentences, making it one of the largest open-source corpora for Chinese-English translation tasks.

- **Corpus Characteristics**: A large number of sentence pairs, covering a wide range of topics, making it suitable for pretraining large language models or fine-tuning specific tasks.
- **Data Domains**: Includes various fields such as news, technology, education, and healthcare, thus covering a wide range of translation task requirements.

**3. Data Format**

- **Storage Format**: Plain text files, separated into source language files (Chinese) and target language files (English).
- **Sentence Pair Alignment**: Each sentence pair is aligned line by line, ensuring translation consistency. Example:

**Source Language File (Chinese)**:
“我们要通过技术创新推动农业发展。”

**Target Language File (English)**:
"We need to promote agricultural development through technological innovation."

- **Data Preprocessing**: Text has undergone standardization and deduplication processes, retaining necessary contextual information.

**4. Data Characteristics**

- **Diversity**: The dataset covers a wide variety of content, including formal documents, web text, and Wikipedia entries, which supports translation tasks across many domains.
- **High Quality**: The sentence pairs have a high degree of semantic alignment, verified by professional alignment algorithms and manual checks, making it suitable for high-quality translation training.
- **Multi-Granularity**: Includes both short texts (e.g., titles) and long texts (e.g., news articles), catering to different translation task requirements.

**5. Applicable Tasks**

The WMT Chinese-English Machine Translation Training Corpus is mainly used for the following tasks:

- **Machine Translation Model Training**: Used for pretraining Chinese-English translation models, supporting deep learning architectures such as Transformer, RNN, and CNN.
- **Machine Translation Model Evaluation**: Provides high-quality aligned texts for evaluating models using metrics like BLEU, METEOR, and ROUGE.
- **Cross-Lingual Text Analysis**: Supports tasks like cross-lingual sentiment analysis and text summarization.
- **Low-Resource Translation Transfer Learning**: Uses the Chinese-English sentence pairs for transfer learning on other language translation models.

**6. Download and Usage**

- **How to Obtain**:
  The dataset is available for free download on the ModelScope platform.
- **Usage Restrictions**:
  Users must comply with the ModelScope platform's usage agreement, and it is prohibited to use the dataset for commercial purposes or illegal activities.

**7. Dataset Advantages**

1. **Large Scale**: Provides a rich corpus of sentence pairs, suitable for large-scale model training.
2. **Multiple Scenarios**: Supports various application scenarios, including news translation, dialogue translation, etc.
3. **High-Quality Alignment**: The alignment accuracy of the sentence pairs is high, making it directly usable for deep learning tasks.

**8. Dataset Limitations**

1. **Noise**: Some web-crawled data may contain noise, requiring further cleaning and processing.
2. **Domain Bias**: The corpus mainly focuses on general domains, and translations in specialized fields may be underrepresented.

**Summary**

The WMT Chinese-English Machine Translation Training Corpus is one of the core resources in the field of machine translation. It is not only large in scale and diverse in coverage but also has high-quality semantic alignment. Researchers can effectively improve machine translation model performance using this dataset, and it also supports research on multimodal translation tasks.
