# Supplementary material for: The intelligent evaluation model of the English humanistic landscape in agricultural industrial parks by the SPEAKING model: From the perspective of fish-vegetable symbiosis in new agriculture
Source: PLoS One. 2025 Jul 16;20(7):e0325332. doi: 10.1371/journal.pone.0325332 (PMC12266420; doi:10.1371/journal.pone.0325332)
Supplement: Attachment — Submitted filename: REVIEWER COMMENTS- plos.docx [file pone.0325332.s002.docx]

**REVIEWER COMMENTS**

Author has done a ground breaking research.

Hypothesis and Research Finding validate each other.
